# The risk of pathological fractures after intralesional curettage in Atypical Cartilaginous Tumours: A retrospective cohort study

**DOI:** 10.1016/j.jbo.2025.100731

**Published:** 2025-11-28

**Authors:** C.H.J. Scholte, K. van Langevelde, D.M.J. Dorleijn, M. Fiocco, D. Broekhuis, P.D.S. Dijkstra, M.A.J. van de Sande, R.J.P. van der Wal

**Affiliations:** a Department of Orthopedics, Leiden University Medical Centre, 2333 ZA Leiden, the Netherlands; b Department of Radiology, Leiden University Medical Centre, 2333 ZA Leiden, the Netherlands; c Department of Orthopedics, University Hospital Ghent 9000 Ghent, Belgium; d Mathematical Institute, University of Leiden 2333 CC Leiden, the Netherlands

**Keywords:** ACT, Bone Tumours, Oncology, Fractures, Chondrosarcoma

## Abstract

**Purpose:**

Atypical cartilaginous tumors (ACT) are locally aggressive, cartilage-producing neoplasms, traditionally classified as chondrosarcoma grade 1. They exist within the spectrum of cartilaginous tumors, between enchondroma and high-grade chondrosarcoma. The diagnosis of ACT remains challenging, with limited consensus on optimal treatment. Despite ongoing debate, intralesional curettage is commonly performed, though the risk of pathological fractures post-treatment remains a significant complication. This study aims to investigate the impact of cortical window design on fracture risk following curettage for ACT in the distal femur.

**Methods:**

We compared two types of cortical windows: smooth oval (low stress riser) and windows with sharp or predrilled rounded corners (high stress riser). A retrospective cohort of 153 patients treated between 2000 and 2020 was included. Fracture occurrence was recorded, and logistic regression models were used to assess the relationship between surgical technique and fracture risk. We corrected for void filling and tumour size.

**Results:**

Our findings revealed no significant difference in fracture risk between the two types of cortical windows (OR = 1.62, 95 % CI:0.66–3.96). However, the use of cement as a void filler significantly increased fracture risk, with odds ratio (OR) approximately five times higher than those using bone chips (OR = 5.00, 95 % CI:1.81–13.88). Tumor size (cm) was also associated with fracture risk (OR 1.31, 95 % CI:1.04–1.63).

**Conclusion:**

The study highlights the importance of choosing appropriate void filling material, with bone chips offering a lower fracture risk compared to cement. These results suggest that optimizing surgical techniques, particularly the choice of filling material, may reduce post-curettage fracture risk in ACT treatment.

## Highlights

• 153 patients with ACT of the distal femur were treated by intralesional curettage.
• No significant difference in fracture risk between cortical window types (OR 1.62, 95% CI 0.66–3.96).
• Cement filling increased fracture risk vs. bone chips (OR 5.00, 95% CI 1.81–13.88).
• Larger tumors were associated with higher fracture risk (OR 1.31 per cm, 95% CI 1.04–1.63).
• Bone chips appear safer void fillers, potentially lowering post-curettage fracture risk.

## 1 Introduction

A central atypical cartilaginous tumour (ACT) is a locally aggressive, hyaline cartilage producing neoplasm arising in the medulla of the appendicular skeleton. Previously classified as grade 1 chondrosarcoma (CS1), ACT is now recognized as a distinct entity due to its locally aggressive behaviour yet very limited metastatic potential. ACT exists within the spectrum of cartilaginous tumours, positioned between enchondroma and high-grade chondrosarcoma [[1], [2], [3], [4], [5], [6], [7], [8], [9], [10]]. The diagnosis of ACT remains challenging both histologically and radiologically, with ongoing global debate in optimizing treatment strategies.

Current diagnostics rely on a combination of imaging, histology, and molecular markers. Radiographically, ACT exhibits endosteal scalloping, calcifications, and may show growth over time; whereas enchondromas typically present as well-defined lesions without interaction with the cortex. Histologically, ACT is characterized by increased cellularity and mild cytologic atypia, but lacks the aggressive features seen in high-grade chondrosarcomas. Molecularly, IDH1/IDH2 mutations are commonly found in both entities, but no definitive biomarker exists to reliably distinguish them [5].

Despite the absence of an international consensus on optimal treatment, intralesional curettage with local adjuvant therapy remains a widely performed approach. Other strategies include wide or marginal surgical (en-bloc) resection for larger or recurrent tumours and a wait-and-scan approach in selected cases [[11], [12], [13], [14]]. The scope and site of the treated lesions varies widely. One of the most significant complications following curettage is a pathological fracture, particularly in the femur [15]. This is in contrast to the wait-and-scan cohort where no pathological fractures occurred [29].

The technique of curettage, type of cortical window created and cavity filling may influence fracture risk, yet no clear guidelines exist [12,[16], [17], [18], [19]].

Curettage is commonly conducted through a handmade cortical window, which can be created in several ways. A commonly used technique is to connect small drill holes in a circular pattern or use a saw to create a rectangular window with sharp or predrilled rounded corners. An alternative technique is to create a smooth oval window with a thin milling tool or high-speed burr. All cortical window types create a stress riser in the cortical bone, which potentially makes the bone vulnerable for a fracture.[20] The risk factors for a pathological fracture after ACT curettage of the distal femur have not been well established, but the type of cortical window might be of significant influence.

**Fig. 1:**
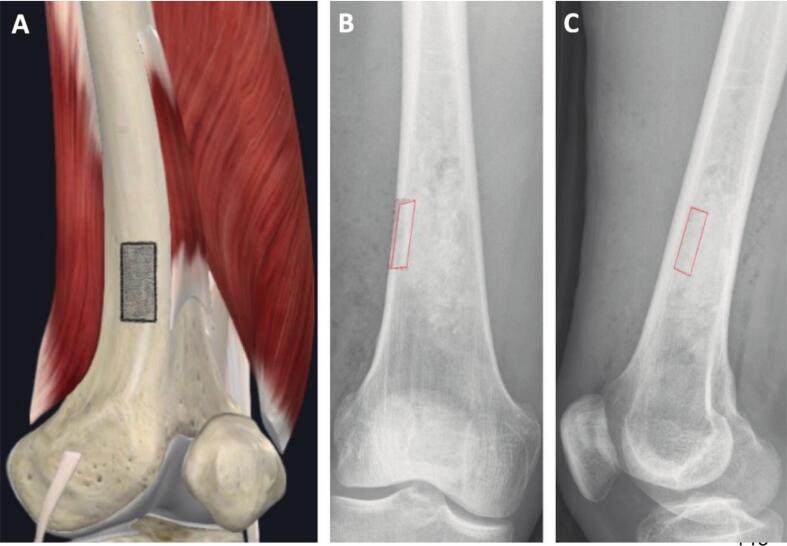
High-stress Cortical Window in the Distal Femur: Anatomical Illustration (A) with AP (B) and Lateral X-ray (C). The cortical window is aligned in red. Image A courtesy of Complete Anatomy. (For interpretation of the references to colour in this figure legend, the reader is referred to the web version of this article.). (For interpretation of the references to colour in this figure legend, the reader is referred to the web version of this article.)

**Fig. 2:**
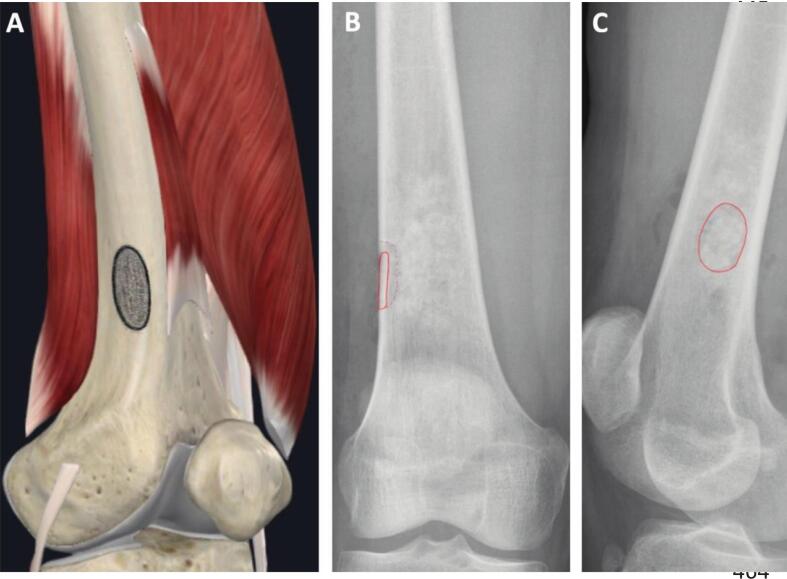
Low-stress Cortical Window in the Distal Femur: Anatomical Illustration (A) with AP (B) and Lateral X-ray (C). The cortical window is aligned in red. Image A courtesy of Complete Anatomy. (For interpretation of the references to colour in this figure legend, the reader is referred to the web version of this article.). (For interpretation of the references to colour in this figure legend, the reader is referred to the web version of this article.)

We compared two types of cortical windows: smooth oval windows (“low stress risers”) (Fig. 1) and windows with sharp or predrilled rounded corners (“high stress risers) (Fig. 2). Based on fracture mechanics and stress concentration theory, we hypothesized that smooth oval windows distribute stress more evenly, reducing fracture risk, whereas sharp or predrilled windows create localized stress peaks, increasing the likelihood of fractures [20,21].

The curettage cavity can be filled or left empty. Common techniques include the use of PMMA bone cement or cancellous bone graft [12,[16], [17], [18], [19]].

In this study we assessed patients with ACT in the distal femur treated with intralesional curettage and the impact of surgical techniques on post-curettage fracture risk.

## 2 Materials and methods

Patients undergoing intralesional curettage for an ACT occurring in the long bones from 2000 to 2020 in a tertiary referral center in the Netherlands were enrolled in the study. Inclusion criteria were histologically confirmed central ACT located in the distal femur (diaphysis or metaphysis) treated by curettage, local adjuvant treatment and allograft bone impaction or PMMA filling. Patients were excluded if they had Ollier’s disease or Maffucci syndrome. All lesions were histologically classified according to the published World Health Organization 2020 consensus criteria. Radiological and histological analyses were performed by experienced musculoskeletal radiologists and pathologists at our centre according to this standard [5]. Minimum follow-up time had to be two years.

Surgery was performed by an oncological orthopaedic surgeon, by an oncological trained fellow alone or under supervision of an oncological orthopaedic surgeon.

Preoperatively, the precise tumour location was identified on MRI, intra-operative fluoroscopy was used to identify to exact tumour location. Using lateral approach to the distal femur, a cortical window was created halfway the length of the tumour. Two different types of cortical windows were created within the studied time: depending on the preference of the surgeon at that time. In the “High Stress Riser” (HSR) group the window was created with an oscillating saw or a chisel which connected the sharp or predrilled rounded corners. In the “Low Stress Riser” (LSR) group an oval window was created with a Lindemann fraise (Conmed linvatec). With this mill, two semi circles of drill holes facing each other are made in the cortex and connected to each other. No corners were created with this technique. This window has an oblong figure, often referred to as an oval. The tumour was then removed macroscopically with use of curettes. Fluoroscopy was used to check for remaining calcified cartilage and measure the full length of the curetted defect. A solution of 85 % phenol (Liquid Phenol, Ph Ned Ed VI quality; BUFA Bv, Pharmaceutical Products, Uitgeest, The Netherlands) was applied for a period of one minute to the interior of the remaining bone cavity with a surgical swab. The phenol was subsequently rinsed out with 96 % ethanol solution followed by saline rinsing. Finally, the bone cavity was filled with deep-frozen non-irradiated impacted allograft cancellous bone chips derived from femoral donor heads (ETB BISLIFE, Haarlem, The Netherlands) or with bone cement (PMMA). During surgery, the bone window was cleaned and submerged in a phenol solution and then rinsed with ethanol and saline and used to reconstruct the cortical defect. After six weeks of non-weight bearing, full weight bearing was gradually established in the next six weeks.

Initially, bone graft was used in combination with a HSR window in our center. However, after a significant number of fractures observed, the approach was revised based on internal evaluation, leading to the use of PMMA cement and a LSR window. As the fracture risk remained high, the decision was made to incorporate a plate for fracture prevention and use bone graft again.

Patient, surgical and tumour characteristics such as sex, BMI and age at the time and type of surgery were collected from medical records, including postoperative radiographs. Fracture occurrence, tumour and treatment characteristics were also analysed: type and length of cortical window (on postoperative radiographs), cavity void filling (cement / bone chips), prophylactic plate fixation, and location measured as the distance from the most caudal point of the cortical window to the knee joint line (measured on first postoperative radiographs).

### 2.1 Statistical analysis

Categorical data are presented as frequencies and percentages; for continuous data summary statistics of mean and standard deviation were presented.

In case of deviation from normality, the median was used as measure of central tendency. Logistic regression models were estimated to study the impact of the type of cortical window on pathological fractures, void filling (cement versus bone chips) and tumour size were incorporated in the models. Variables were chosen based on clinical knowledge and previous studies [[22], [23], [24], [25]]. Odds ratios (OR) with 95 % confidence intervals (CI) are reported.

### 2.2 Patients with plate fixation were excluded from the analysis

Statistical analysis were performed with IBM SPSS Statistics v.25 (IBM, USA).

### Ethical approval

This study was approved by the Medical Ethical Review Committee of Leiden, the Hague, and Delft.

## 3 Results

### 3.1 Patient flow

**Fig. 3:**
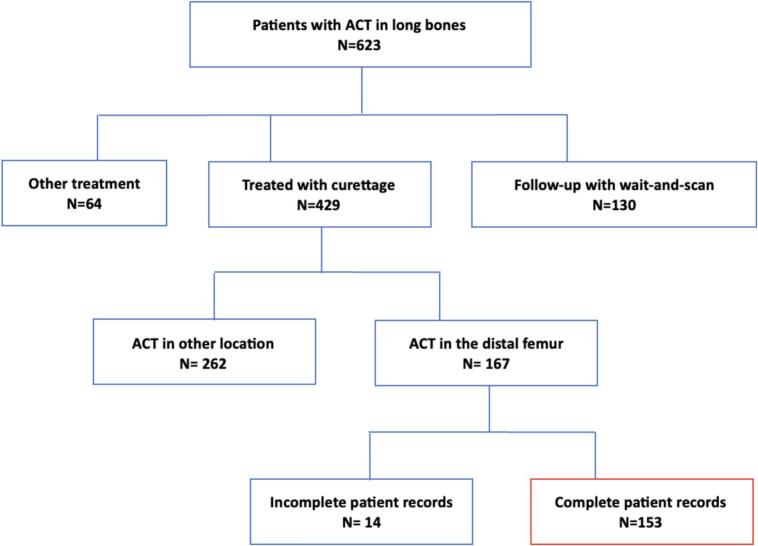
Flow diagram of study participants.

In total, 623 patients were screened for eligibility (Fig. 3). 130 patients were followed-up through wait-and-scan, where patients are under active surveillance with MRI and are not treated surgically. 64 patients underwent other treatment such as RFA or en bloc resection. In the final five years of this study, most of these cases were treated non-operatively, aligning with a shift in the treatment approach towards a wait-and-scan policy as the preferred treatment strategy in our center. For a short period and limited to small lesions (<2.5 cm), RFA was applied. This left a total of 429 patients treated with curettage of whom 167 were in the distal femur. In 14 patients, the medical records were incomplete, precluding their inclusion. One fracture was present in this group. This left a total of 153 patients for complete case analysis.

### 3.2 Baseline characteristics

The baseline characteristics are displayed in Table 1. The studied group consisted of 99 (65 %) females, with a mean age of 52 +/- 11 SD and a mean BMI of 27 +/- 5 SD (Table 2).

**Table 1:** Patient’s characteristics at baseline.

Total	153
Mean age at the time of surgery (SD)	52 (11)
Female (%)	99 (65)
Mean BMI (SD)*	27 (5)
ACT size in mm (%)	
≤20	6 (4)
21–50	79 (52)
51–100	62 (40)
101–150	5 (3)
≥150	1 (1)

*SD = standard deviation

**For the value BMI, out of 153 cases 29 had missing data.

**Table 2:** Fracture Distribution in relation to patient and tumour characteristics.

Variable	Category	No fracture	Fracture	Percentage fracture per group (%)
Sex	Female	83	16	16
	Male	42	12	22
Plate fixation	No	91	25	22
	Yes	34	3	8
DTC (mm)*	0–50	36	2	5
	51–100	42	11	21
	101–150	36	10	22
	>150	11	5	31
Age	0–50	66	0	0
	51–70	59	16	21
	>70	0	12	100
Void villing	Cement	21	13	38
	Allograft bone chips	104	15	13
BMI	<18	0	0	0
	18–25	19	8	30
	25–30	53	12	18
	>30	27	5	16
	Missing	26	3	10
Tumour size CC **(mm)	<26	17	0	0
	26–50	57	11	19
	51–75	30	12	29
	76–100	15	5	25
	>100	6	0	0
Tumour size CC (mm)**	≤40	49	7	13
	>40	76	21	22
Cortical window size (mm)	<20	51	10	16
	21–30	59	10	15
	31–40	13	8	38
	>40	2	0	0
Cortical window type	High stress riser	34	13	28
	Low stress riser	91	15	14

*Cut-off based on clinical experience.

*DTC = distance from inferior border of cortical window to cortex of femoral condyle.

^**^CC = craniocaudal.

^***^Cut-off based on BACTIP-criteria^2^.

### 3.3 Fracture distribution

**Fig. 4:**
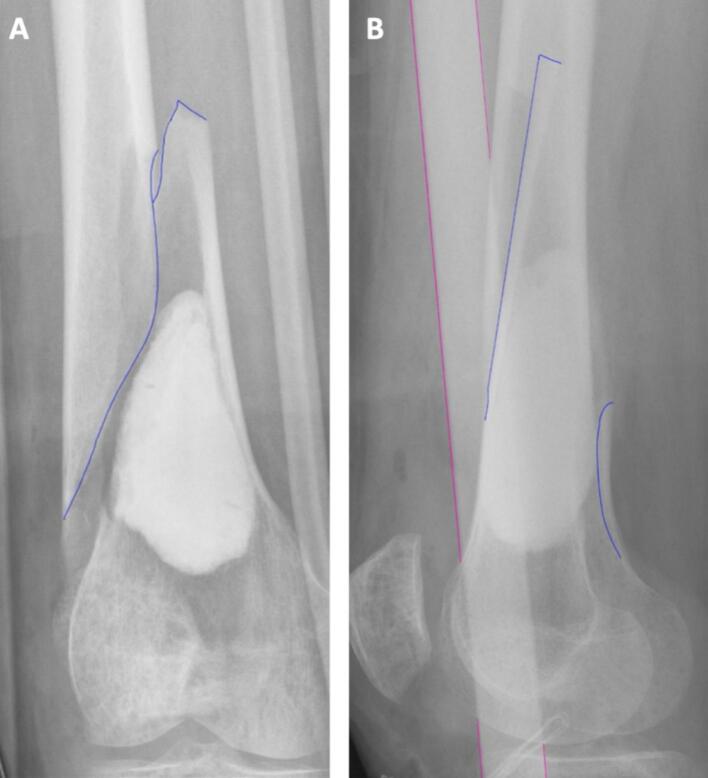
Anterior posterior (AP) (A) a lateral X-ray images (B) of a fracture (blue line) after curettage of an ACT with cement void filling. The pink line indicates the splint. (For interpretation of the references to colour in this figure legend, the reader is referred to the web version of this article.). (For interpretation of the references to colour in this figure legend, the reader is referred to the web version of this article.)

**Fig. 5:**
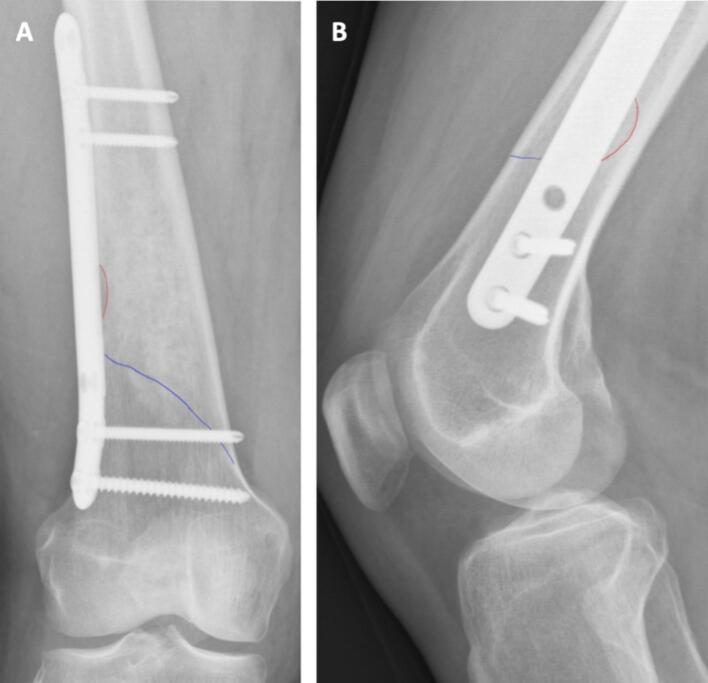
AP (A) and lateral X-ray images (B) of a fissure (blue line) after curettage with prophylactic plate fixation. The fissure occurred despite the presence of the plate fixation. The red line indicates the oval shaped cortical window. (For interpretation of the references to colour in this figure legend, the reader is referred to the web version of this article.). (For interpretation of the references to colour in this figure legend, the reader is referred to the web version of this article.)

In table 2 the distribution of fractures is shown. In total 28 fractures occurred. Cortical window characteristics varied, with fractures occurring in 28 % of patients with high-stress windows compared to 14 % in patients with low-stress windows. The number of fractures also varied with the distance from the cortical window to the knee joint line. Fractures were seen in 5 % of cases with a distance of 0–50 mm, increasing to 21 % at 51–100 mm and 22 % at 101–150 mm. At > 150 mm from the joint line, 31 % of patients sustained a fracture. Among different age groups, no fractures were observed in patients under 50 years, while 21 % of patients aged 51–70 years and all patients over 70 years sustained fractures. Fractures occurred in 38 % of cases treated with cement and 13 % of cases treated with allograft bone chips. Fig. 4 provides an example of a fracture after curettage with cement. Among tumours larger than 40 mm, fractures were seen in 22 % of cases versus 13 % below 40 mm. Patients with prophylactic plate fixation had a fracture rate of 8 % (N = 3). However, these were only fissures with no clinical consequences, as they required no further treatment. Fig. 5 provides an example of a fracture in a femur with prophylactic plate fixation.

Table 3 and Table 4 show a subgroup analyses, excluding patients treated with prophylactic plate fixation. Table 3 describes the distribution of fractures, based on cortical window stress and void filling material. In the HSR group (n = 45), fractures were observed in 8 out of 35 cases (23 %) treated with bone chips and in 4 out of 10 cases (40 %) treated with cement. In the LSR cortical window group (n = 71), fractures occurred in 9 out of 23 cement-treated cases (39 %) and in 4 out of 48 cases treated with bone chips (8 %).

**Table 3:** Fracture distribution based on Cortical Window type and Void Filling Material in ACT patients treated without prophylactic plate fixation.

**Cortical window**	**Void filling**	**Fracture**	**No fracture**	**Total**
HSR*	Bone chips	8	27	35
	Cement	4	6	10
LSR**	Bone chips	4	44	48
	Cement	9	14	23

*HSR = High Stress Riser.

**LSR = Low Stress Riser.

**Table 4:** Fracture distribution based on Tumour Size and DTC in ACT patients treated without prophylactic plate fixation.

**Tumour size**	DTC*	**Fracture**	**No fracture**	**Total**
≤ 4 cm	≤ 5 cm	2	26	28
	> 5 cm	5	18	23
> 4 cm	≤ 5 cm	0	8	8
	> 5 cm	18	39	57

*DTC = distance from inferior border of cortical window to cortex of femoral condyle.

Table 4 categorizes patient by tumour size and the distance from the cortical window to the caudal cortex of the femur condyle. In cases with tumours ≤ 4 cm (n = 51), fractures were recorded in 2 out of 28 patients with a cortical window distance of ≤ 5 cm and in 5 out of 23 patients with a distance > 5 cm. Among patients with tumours > 4 cm (n = 65), fractures were only recorded when the cortical window was more than 5 cm from the condyle (18 out of 57 cases), while no fractures were seen in cases with a distance ≤ 5 cm (0 out of 8 cases).

### 3.4 Logistic regression models

A univariate logistic regression model was estimated to study the association between cortical window shape and fracture risk. There was no association between the covariate and outcomes (OR = 1.62, 95 % CI: 0.66–3.96, reference category LSR window). After adjusting for void filling and tumour size (Table 5), the odds ratio for cortical window shape was equal to 1.62 (95 % CI: 0.59–4.40, reference category LSR window). The use of cement as void filling material was associated with an increased risks of fracture (OR = 5.00, 95 % CI: 1.81–13.88, reference category allograft bone chips). Tumour size was associated with fracture risk (OR = 1.31, 95 % CI: 1.04–1.63). This OR indicates a statistically significant increase in risk of fracture by 30 % for each 1 cm increase in tumour size.

**Table 5:** Multivariate logistic regression for cortical window shape on fracture risk.

**Variable**	Odds Ratio	95 % Confidence Interval
Cortical window High Stress Riser Low Stress Riser	1.62 Ref.	0.59–4.40
Void filling Cement Allograft bone chips	5.00 Ref.	1.81–13.88
Tumour size (cm)	1.31	1.04–1.63

## 4 Discussion

The findings of this study provide important insights into the risk factors for pathological fractures following curettage followed by adjuvant treatment of ACT in the distal femur. Our initial hypothesis was that the type of cortical window would significantly influence fracture risk. However, our results indicate that, while there are trends suggesting an effect, the impact of cortical window shape was not large or significant when anticipated confounders were considered. In contrast, the type of void filling showed a significant association with fracture risk, with the use of cement posing a substantially and statistically significant higher fracture risk (OR = 5.00, 95 % CI: 1.81–13.88). Tumour size also showed a small significant association with fracture risk (OR 1.31, 95 % CI: 1.04–1.63).

Void filling was chosen as a correcting factor because previous studies have highlighted that cement can lead to stress shielding, increasing the odds of fractures in weight-bearing bones such as the femur.[22,23] Also, tumour size was chosen as a correction factor because literature shows that fracture risk increases with advancing tumour size.[24,25] Moreover, a larger tumour results in a bigger defect, which in turn requires more cement for filling. This could further amplify the effects of cement on bone mechanics. Variables were chosen based on clinical experience. The size of the defect did not play a role in the selection of void filling.

This study describes the largest group cohort in literature of patients with ACT of the distal femur treated with curettage followed by local adjuvant treatment. In the literature fractures after intralesional curettage are mentioned as a heterogeneous group with ACT’s in various locations. Frequently, other tumours were included as well. To determine the pathological fracture incidence and risk factors by using different cortical bone windows, we only included patients with a surgical treated ACT in the distal femur, without cortical breach.

### 4.1 Interpretation of finding

Although our data shows a small effect, results from the logistic regression analysis does not provide statistically significant confirmation of our hypothesis that smooth oval cortical windows function as 'low stress risers' by distributing mechanical stress more evenly across the cortical bone. However, a key finding in our data is that the type of void filling material has a significant impact on fracture risk. Our results show that bone chips seem a better option in preventing fractures, compared to PMMA cement. A possible explanation is that bone chips integrate biologically with the surrounding bone over time, whereas cement remains a rigid filler that does not contribute to bone remodelling.

PMMA creates an abrupt transition to softer bone, and this stiffness may act as a stress riser. Additionally, the thermal effect of bone cement may play a role. The exothermic polymerization of PMMA can induce thermal injury to the cortex, which may weaken the bone. Comparable effects have been observed in other setting where abrupt temperature changes occurred. For example, extreme cooling has also been shown to cause cortical damage and weakening of the surrounding bone. These observations suggest that extreme thermal shifts may contribute to postoperative fracture risk [22,26,31]. It should also be noted that cement carries other possible limitations described in the literature. The exothermic polymerisation may also contribute to secondary osteoarthritis under certain conditions, particularly in proximity to the joint surface. However, this is rarely the case [34].

Three out of 37 patients with prophylactic plate fixation experienced a fissure with no further clinical consequences. It should however be noted that plate fixation also has disadvantages including a longer surgery time, increased infection risk, higher costs, and the potential need for plate removal [27,28].

### 4.2 Comparison with other studies

Our findings are consistent with prior research on mechanical stress distribution in bone defects and the impact of window design on structural integrity. However, previous studies on ACT treatment have primarily focused on oncological outcomes.(13, 18, 19, 29) While some studies have acknowledged the risk of fractures post-curettage, our study is one of the first to systematically compare different cortical windows designs and filling materials.

Previous studies have highlighted that cement can lead to stress shielding due to the thermal effect, increasing the risk of fractures in weight-bearing bones such as the femur [22,23]. This temperature effect can also be seen when instead of heat cold temperatures are applied such as in cryoablation, which has been associated with an increased risk of fractures [30,31].

## 5 Limitations

Our study has several limitations. Firstly, the retrospective nature of this study makes it prone to incomplete records and selection bias. Due to the relatively small sample size and the limited number of events in our dataset, the number of variables for our logistic regression that could be included in the model was restricted. Given the low event rate in our study, we had to be cautious in selecting variables, prioritizing with the strongest theoretical relevance. As certain potentially relevant factors could not be included, this limitation may have impacted the comprehensiveness. Factors such as BMI, tumour location, and the size of the cortical window have not been included in the analysis. Literature suggests that cortical window length may also have an influence on fracture risk [32]. Future research with larger datasets and a higher number of events could allow for a more detailed multivariable analysis. Another limitation of this study is that patient adherence to weight-bearing restrictions varies in real life, affecting fracture risk. Factors like therapy compliance, cognitive ability (e.g., IQ), lifestyle, and overall bone quality also play a role but were not included in this analysis. The importance of these factors has been shown in previous studies [26,33].

## 6 Conclusions

This study highlights the critical role of void filling material in fracture risk following curettage of ACT in the distal femur. Our study shows a reduction in fracture risk when using bone chips, providing valuable insights for optimizing surgical techniques in the treatment of ACT. Furthermore, cortical window shape may influence fracture risk.

## 8 Consent to participate

Not applicable.

## 9 Consent for publication

Consent for publication was obtained.

## 10 Availability of data and material

Data and materials are not available.

## CRediT authorship contribution statement

**C.H.J. Scholte:** Conceptualization, Data curation, Formal analysis, Investigation, Methodology, Software, Validation, Visualization, Writing – original draft, Project administration. **K. van Langevelde:** Writing – review & editing, Visualization, Supervision. **D.M.J. Dorleijn:** Writing – review & editing, Supervision, Methodology. **M. Fiocco:** Validation, Methodology, Formal analysis. **D. Broekhuis:** Writing – review & editing. **P.D.S. Dijkstra:** Writing – review & editing, Conceptualization. **M.A.J. van de Sande:** Writing – review & editing, Supervision, Resources, Conceptualization. **R.J.P. van der Wal:** Writing – review & editing, Conceptualization.

## Ethics approval

The study was approved by the appropriate ethics committee (please insert name of the committee and approval number).

## Funding

No funding was received for conducting this study.

## Declaration of competing interest

The authors declare that they have no known competing financial interests or personal relationships that could have appeared to influence the work reported in this paper.
